# Atomistic Simulations Decode the Mechanisms of DNA and RNA Processing Enzymes: Function through Motion

**DOI:** 10.1021/acs.accounts.6c00391

**Published:** 2026-07-16

**Authors:** Marco De Vivo

**Affiliations:** Molecular Modeling and Drug Discovery Lab, 121451 Istituto Italiano di Tecnologia, Via Morego 30, 16163 Genoa, Italy

## Abstract

Enzymes and nucleic acid processing machines are among the most sophisticated molecules evolved by nature. These include several vital enzymes such as polymerases, nucleases, topoisomerases, and RNA enzymes. These enzymes exhibit complex structures and multicomponent assemblies that enable an exceptional efficiency and specificity in carrying out the fundamental chemical reactions required for life. Yet, these structures pose a challenge to our understanding of their catalytic precision, as this requires atomistic insight into how they operate on DNA or RNA. Indeed, the static or quasi-static view from experiments fails to fully explore the conformational space that these enzymes traverse during catalysis. Thus, advanced molecular simulation techniques are increasingly integrated with experimental results to overcome such sampling limitations and to explore the configurational space, thereby defining the functional dynamics of structural motions and rearrangements that would otherwise remain unclear.

In this Account, I will examine how atomistic multiscale molecular simulations and free energy calculations—recently coupled with AI-guided enhanced sampling methods—have contributed to clarifying enzymatic mechanisms that could only be hypothesized by examining structures, as evidenced by increasingly complex structural biology results. This is a time when we can simulate ultralarge realistic model systems comprising proteins, nucleic acids, ions, and water molecules, all of which are critical components for enzymatic function. Remarkably, such modeling and simulations can nowadays enable the prospective exploration of increasingly structurally complex systems.

In this scenario, there is today a relevant body of work that defines function through motion as a key element in these enzymes. My group has generated a broad collection of results on a range of nucleic acid processing enzymes, from polymerases to nucleases and RNA enzymes, which altogether highlight how *dynamics define function*, with specific residues—often evolutionarily conserved and localized in the second coordination shell of the catalytic core—that are strategically positioned to operate in a cooperative and highly coordinated fashion for specific enzymatic actions on nucleic acids. These coordinated residue motions, near the reaction center, appear distinct from large allosteric movements of distal domains. Instead, they resemble finely tuned, small-scale mechanisms that marry complexity with the precise, clocklike efficiency evolution has built into the catalytic core. How such elaborate enzymatic architectures *move-to-function*, captured and demonstrated by the most recent computational work and structural data, will therefore be the core topic discussed in this Account. While the concept of “*function through motion”* can obviously be extended to other catalytic systems, what is particularly remarkable about these enzymes—even if not unique—is that their functional complexity is handled with extraordinary accuracy through precise motions, despite the system’s architectural complexity, on the move. These recent mechanistic findings also provide insights into how to engineer or modulate these enzymes, with implications for several scientific activities centered on nucleic acid chemistry.

## Graphical abstract

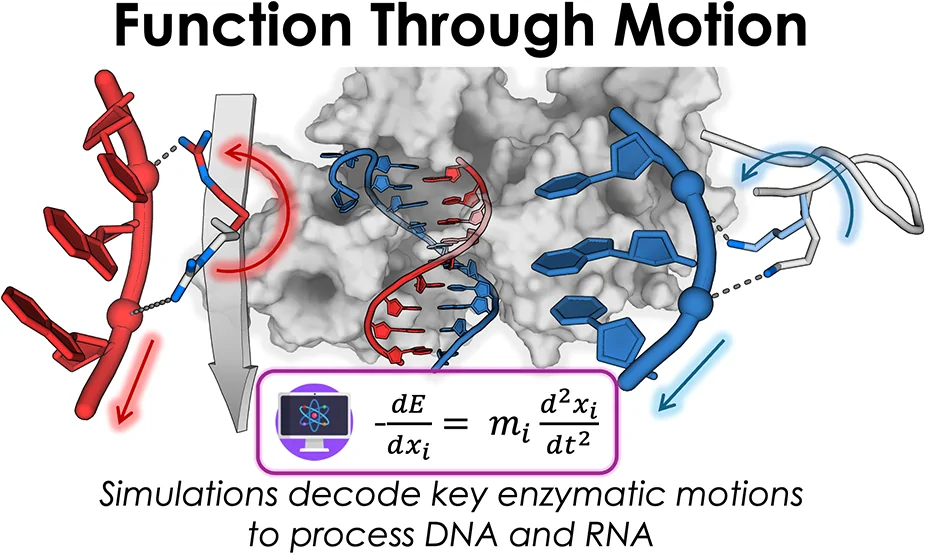

## Key References

-

Martino, G. ; Manigrasso, J. ; La Sala, G. ; Marcia, M. ; De Vivo, M. Controlled Dynamic Remodeling of the Spliceosome Active Site Enables the First Step of Splicing. Proc. Natl. Acad. Sci. U. S. A. 2026, 123­(1), e2522293123.41474748 10.1073/pnas.2522293123PMC12773743 Our equilibrium and enhanced-sampling simulations elucidate how a minimal set of positively charged residues near the catalytic center dynamically remodels the megadalton spliceosome active site during the initial splicing step.^1^
-

Visigalli, E. ; Trizio, E. ; Bonati, L. ; Vidossich, P. ; Parrinello, M. ; De Vivo, M. Coordinated Residue Motions at the Enzyme-Substrate Interface Promote DNA Translocation in Polymerases. J. Am. Chem. Soc. 2025, 147, 22972–22985.40527759 10.1021/jacs.5c05888PMC12232177 This work utilizes deep-learning-guided enhanced-sampling simulations to decode the coordinated motion of specific residues that enable the asynchronous translocation mechanism along DNA strands in certain polymerase enzymes.^2^
-

Manigrasso, J. ; Chillón, I. ; Genna, V. ; Vidossich, P. ; Somarowthu, S. ; Pyle, A. M. ; De Vivo, M. ; Marcia, M. Visualizing Group II Intron Dynamics between the First and Second Steps of Splicing Nat. Commun. 2020, 11, 2837.32503992 10.1038/s41467-020-16741-4PMC7275048 Our equilibrium and enhanced-sampling simulations demonstrate that precise residue motions at the catalytic site of group II introns are crucial to the splicing mechanism. We also propose that a similar enzymatic strategy operates at the spliceosome catalytic site.^3^

## Introduction

Enzymes and nucleic-acid–processing machines represent some of the most refined molecules produced by evolution. For example, these include polymerase enzymes that copy DNA and RNA.^4,5^ Nuclease enzymes are capable of cutting nucleic acids, which is a necessary process for gene editing.^6^ Topoisomerase enzymes can crucially control DNA topology in cells.^7^ One of the most complex enzymatic machines, i.e., the human spliceosome, is a megadalton structure that encloses RNA strands, multiple proteins, and numerous cations to form a complex and dynamic enzymatic architecture to catalyze splicing reactions, a vital process for gene expression.^8^ Remarkably, these enzymes exhibit complex structural architectures that display extraordinary efficiency and specificity in performing some of the most fundamental chemical reactions essential for life. That is, these fundamental machines are of primary biological and pharmacological relevance, whose understanding has numerous scientific implications, including the discovery of new disease treatments.^9–11^

The efficiency of such complex enzymatic machines, however, does not only emerge from their static, multicomponent complex structures, as defined by structural data and empirical mutagenesis and biophysical experiments.^5,6,8^ Nowadays, there is overwhelming experimental evidence that their molecular function primarily emerges from major conformational fluctuations and dynamical processes that define catalysis as a multistate energy landscape.^12–14^ For example, high-resolution structures have revealed that specific polymerases, RNA enzymes, or the human spliceosome require the coordinated molecular dynamics of specific structural determinants for function.^1–3^ In other words, these experimental results underscore that the catalytic function of such complex machines is closely tied to their precise dynamic motion, which cannot be inferred solely from their static or quasi-static representations, complex molecular composition, and overall structural assembly. As a consequence, the rational design and modulation of such enzymes remain difficult when their catalytic efficiency, selectivity, or regulation depend on crucial, yet rare, and transient dynamical motions that are poorly understood.

Over the past decade, however, structural biology has been increasingly and successfully coupled with extensive multiscale molecular simulations, enabling us to profoundly shape our understanding of enzymatic catalysis and nucleic acid processing.^15–17^ In this Account, I discuss how the precise dynamics of specific components of these enzymatic machines have been captured and elucidated using state-of-the-art atomistic molecular dynamics and enhanced sampling free-energy simulations, integrated with experimental data. In this way, complex yet well-coordinated motions of specific residues, or certain structural motifs in these enzymes, have been demonstrated to be essential for substrate recognition, binding, and catalysis of precise nucleic acid substrates in several significant cases.^1–3^ Ultimately, the integration of advanced simulations and experiments has highlighted the emergent role of the *structure-dynamics-function relationship* in such enzymes, which we found to be precisely the hallmark of enzymatic catalysis and nucleic acid processing.

Through a few selected examples, ranging from second-shell residue motions in polymerases and nucleases to complex structural reorganization in RNA enzymes, such as group II introns, a picture emerges in which the coordinated dynamics of specific enzymatic structural components define function, opening up mechanistic pathways that govern access to reactive states. These coordinated motions of residues in the vicinity of the enzymatic reaction center differ from the large-scale allosteric dynamics of peripheral domains but appear more like finely tuned, miniaturized mechanisms that combine complexity with the remarkable catalytic precision of an evolutionarily engineered timepiece operating at the core of the catalytic machine.

## Key Elements and Considerations for Computational Simulations of Nucleic Acid-Processing Enzymes

Two crucial, interrelated questions must precede the use of atomistic multiscale simulations to elucidate the enzymatic processing of nucleic acids. The first question is *what exact mechanistic question do I want to investigate via atomistic multiscale molecular dynamics*? The second question, which follows, is *which computational approaches and parameter sets are most appropriate for investigating a hypothetical mechanism that often involves complex multicomponent molecular models*? Addressing these key questions helps us appreciate what such simulations can offer and teach us, as discussed in the following paragraphs. In addition, by being aware of the current limitations in the methodological aspects of the study, one can set realistic expectations and correctly position computational investigations for success.

### The Mechanistic Questions Determine the Methodological Approach

Investigations on catalytic steps in which bonds are broken and/or formed clearly require consideration of electrons, thereby implying the use of quantum mechanics (QM).^18–20^ On the other hand, conformational changes and structural reorganizations (that do not involve chemical reactions) can be investigated with molecular mechanics (MM).^21–24^ When these methods are coupled to the equation of motion, we can run either classical molecular dynamics based on MM, which nowadays spans nanoseconds to milliseconds,^16,25,26^ or quantum simulations based on QM, which typically span tens to hundreds of picoseconds.^27,28^ In addition, the hybrid QM/MM approach, coupling QM with MM, is the most commonly used method for investigating (enzymatic) catalysis, with a core of QM atoms at the reactive center embedded in a large MM model, while the whole system is in motion.^27,29–32^ For example, nucleotidyl transfer reactions during polymerase catalysis or phosphodiester bond hydrolysis reactions in nucleases for nucleic acid cleavage are well-suited for investigation with QM/MM simulations, which can be performed at different levels of QM theory.^33–36^ On the other hand, large motions of structural motifs that reorient from reactants to products, or adjust their conformation upon substrate binding, pose mechanistic problems that can be addressed through classical molecular dynamics (MD) simulations.^14,16,25,26^ Classical MD is also widely used to identify long-range allosteric communication pathways in enzymes, with several notable applications to protein-nucleic acid complexes, as well.^14^

Challenges are typically inherent to the accuracy of the parameters used for MM calculations in MD and to the limited configurational sampling reachable by the simulations, whether they are classical or quantum. Thus, accuracy and sampling remain the two primary challenges and sources of error in simulations, each influencing the other as two sides of the same coin that together determine the quality of the resulting findings. When dealing with nucleic acid processing machines, the presence of transient ions can pose an additional challenge, requiring extra care from the user during system setup. These diverse yet complementary computational approaches and related challenges, along with best practices for their application, are thoroughly discussed in several instructive Reviews, including on enzymatic machines acting on nucleic acids.^14,20^

### The Reaction Coordinate for Free-Energy Calculations Is Crucial

Once the most appropriate computational approach for the simulations is determined, depending on the specific questions under investigation, simulations can be coupled with a free-energy estimation method.^37^ To this end, numerous approaches exist for calculating the energetics of the process under study.^37^ To name a few, free energy perturbation (FEP) and thermodynamic integration,^37,38^ umbrella sampling,^39,40^ metadynamics,^41,42^ and several other approaches have been applied to the study of chemical processes in nucleic acid-processing enzymes (as well as in other enzymes).^37,43^ Importantly, most of these methods require the identification of a reaction coordinate (RC, often also termed a collective variable, CV) to be used to calculate the free energy of the process, e.g., moving from one state A (e.g., the reactants) to another state, B (e.g., the products) during catalysis.^41^ In the first approximation, the chosen RC is a minimal set of degrees of freedom of the system that captures the transition from A to B. In the simplest cases, such as an S_N_2 reaction involving a phosphoryl transfer, this can be the distance between the attacking nucleophilic oxygen and the phosphorus atom to be attacked.^44^ Or, in the case of a structural change in a loop, the RC can be set to a selected distance that captures the conformational change under investigation.^45^ While a variety of RCs have been widely used in simulations,^46,47^ complex multistep chemical and physical processes in multicomponent nucleic-acid-processing enzymes remain challenging to model with simple RCs.

Indeed, the identification of the most truthful RC—which is, in most cases, a simplification of the exact and unknown complex RC—remains a challenge, as processes of interest can often involve multiple moving protein residues, transient metal ions, water molecules, and nucleic acid strands on the move during multistep catalytic processes. In this regard, the advent of machine learning-based RCs is particularly timely.^48^ Such complex RCs can incorporate many degrees of freedom (e.g., interatomic distances, angles, coordination numbers, etc.) used as “features” to define complex motions.^49,50^ For example, in this Account, the implementation of such machine-learning-based RC is discussed (*vide infra*) to investigate the complex physical process of enzyme-catalyzed DNA translocation in polymerases.^2^ Nonetheless, one key aspect must be kept in mind when using machine learning-based RCs: While such complex RCs enable observation of the process under investigation, little is self-evident from these transitions about the exact mechanism they capture. That is, the fact that the system transits from A to B, as determined by finely tuned machine-learning-based RCs, does not teach us the enzymatic mechanism, which remains inherently contained within the trajectories. Therefore, the user must critically analyze these trajectories to determine exactly how the dynamical motions of the key components (i.e., the implemented features) are essential for function. This means that the final set of features used to obtain functional machine-learning-based RCs is key to success. The critical analysis of these transitions can yield possible enzymatic mechanisms and identify the mechanistic determinants that enable it to function with exquisite efficiency. Often, however, this step requires translating these complex RCs into more intuitive metrics that help generate comprehensible mechanistic insights.^51^ This aspect remains somewhat dependent on the user’s chemical intuition and experience in selecting the best features, often requiring substantial analytical effort to determine the process’s essential components. The bottom line is that selecting the most appropriate features for generating machine-learning-based RCs and critically interpreting the transitions obtained from enhanced-sampling simulations are key steps toward meaningful mechanistic insights. This procedure, therefore, implies that human oversight remains essential and can significantly influence the final outcome.

### Experimental Data Are Essential for Defining the Outcomes of Mechanistic Studies: Properly Frame Your Research Question before Embarking on Complex Simulations

In addition to all of the aforementioned methodological considerations, there remains one more strategic aspect to stress: *the proper framing of the mechanistic question of interest*. Indeed, what can greatly impact the future outcome of the computational study, thus forcing a cautious choice of the mechanistic problem to tackle, is the proper formulation of the mechanistic question under investigation, meaning that this question is carefully set within a solid experimental data framework that will serve to prove or disprove the computational evidence.^17^ While this may sound trivial and always a wise *modus operandi*, it is indeed where most computational simulations lose their potential value. In fact, complex mechanistic questions, especially dealing with complex multicomponent model systems including proteins and nucleic acids, should be based on a solid experimental data framework that typically centers on multiple structural models (X-ray and/or cryo-EM structures), mutagenesis data, and a set of biophysical measures of substrate affinity, catalytic efficiency, and/or specificity, as they relate to the problem of interest. These are the types of (often already existing) data that, as exemplified in the forthcoming paragraphs and selected examples, must be well integrated with simulations to render the computational evidence robust and credible. At times, correctly configuring this data framework for complex molecular systems can be highly demanding. Yet, this is a critical step for testing and validating *in silico* evidence, especially given the inherently complex nature of the systems under investigation, in which proteins, DNA, RNA, water, and ions move to shape mechanisms that enable extreme precision in catalysis.^52^ Indeed, at other times, it is just not possible to build a solid data framework, rendering the problem unsuitable for (realistic) computational investigations to generate testable mechanistic insights. Without a solid experimental data framework, which should be defined and constructed prior to simulations, these computational efforts on complex systems would be at risk of producing evidence that would likely be considered only speculative and of minimal impact. With this spirit, and with the support of the rapidly growing data set on nucleic acid processing enzymes generated by experimentalists,^15,53,54^ computational simulations are poised to provide increasingly informative prospective explorations of these enzymatic machines,^17^ serving their understanding, regulation, and, eventually, their rational design.

## Role of Local Dynamics at the Core of Polymerase Enzymes

Over the years, atomistic multiscale molecular simulations have been instrumental in examining several aspects of polymerase (Pol) enzymes, either focusing on their multistep catalytic mechanism via QM/MM simulations, or on crucial conformational changes related to their function, investigated using classical MD.^12,55–57^ Starting with their enzymatic mechanism for nucleotide addition, which occurs at the two-metal center catalytic site, several informative studies point to the exact mechanism for metal-aided phosphoryl transfer and nucleotidyl addition at the growing primer, according to the template strand.^58^ Importantly, there are several considerations for this complex multistep reaction mechanism, e.g., how the catalytic 3′-OH is activated, and how the pyrophosphate (PPi) leaving group is eventually released.^33,59–63^ This also implicates the possible involvement of additional transitory metal ions, which can diffuse and be located near the catalytic center to facilitate the chemical step and product release.^12,64–68^

More recently, we have used atomistic multiscale molecular simulations to investigate polymerase fidelity and processivity, two key properties of Pols.^12,69^ Fidelity refers to their ability to incorporate the correct nucleotide into the growing strand during DNA synthesis. On the other hand, processivity is the average number of nucleotides incorporated during a single Pol-substrate binding event. Notably, designing polymerases with tunable fidelity and processivity would offer key practical and conceptual advantages.^12,69^ For example, in PCR or DNA synthesis, very high fidelity is crucial to avoid introducing potentially harmful mutations.^70^ On the contrary, in directed evolution experiments, antibody maturation, or library generation, lower fidelity is desirable, where a predictable mutation rate and spectrum would make the current “error-prone PCR” methods far more efficient and reproducible.^71–73^ Also, Pols that maintain processivity but tolerate lesions without catastrophic stalling could recover more information from challenging samples.^74,75^ Thus, having Pols with programmable fidelity and processivity would transform them from “fixed biological tools” into precision instruments for new experimental designs.^12^

Integrating recent structural and kinetics data of high-fidelity Pol β and low-fidelity Pol η with equilibrium molecular dynamics and free-energy simulations of paired and mispaired reactant complexes of these Pols, we demonstrated that local dynamics at the reaction center determine whether the nucleophile is optimally aligned to incorporate either the correct (dCTP) or incorrect (dATP) nucleotide opposite a template deoxyguanosine (dG).^76^ In Pol β, local structural distortions at the catalytic site are observed only in the dG:dATP mispair complex, energetically disfavoring incorrect nucleotide incorporation and thereby promoting high fidelity. In contrast, Pol η exhibits greater flexibility in base-pair shape complementarity at the catalytic site.^76^ This flexibility allows both matched and mismatched reactive configurations to form with similar ease, explaining the low fidelity of Pol η, consistently with experimental observations.^4,77^ Thus, these simulations helped explain the mechanistic causes for the considerable discrepancy in fidelity between Pol β and Pol η.^76^ In addition, comparisons with other polymerases confirmed that local, metal-mediated structural dynamics at the catalytic site are decisive in modulating Pol fidelity, enriching previous observations and hypotheses from static structural data.^76,78^

We could also highlight how the formation and dynamic stability of specific interatomic interactions around the incoming nucleotide influence the overall architecture of the binding site. In Pol β, this was demonstrated by examining the dynamics of the interaction network formed by residue R283, whose mutant R283A has been experimentally shown to have a lower capacity to differentiate correct (G:dCTP) vs incorrect (G:dATP) base pairing.^68,79^ The side chain of R283 is crucial for the formation of a proper catalytic state, preluding nucleotide addition of the correct vs incorrect nucleotide.^80^ The lack of such a key interaction network in the R283A mutant lowers the ability of Pol β to efficiently discriminate between correct vs incorrect nucleotides in our simulations. This explains why certain Pols’ mutants can significantly alter the local catalytic environment and its dynamics, thereby influencing the selection of correct vs incorrect nucleotides.^79^ A similar dynamical effect on Pol fidelity was observed for processing substates containing the common 8-oxoguanine (8OG) DNA lesion, which can be bypassed by Pols with low catalytic efficiency and high error frequency.^81,82^ Equilibrium MD and metadynamics showed that 8OG consistently adopts different conformations when bound to Pols (Pol β and Pol η), compared to when in isolated DNA. *In silico* mutations of residues interacting with the 8OG phosphate group could alter the preferred 8OG conformations, thereby indicating 8OG conformation as a key factor in the processing of damaged DNA.^82^ Notably, a similar simulation strategy was also helpful to investigate how the glycosyl torsion in 8OG is reflected in Hoogsteen (HG) vs Watson–Crick (WC) base pairs in Pol η, yielding results in excellent agreement with experimental data across all examined DNA systems.^83,84^ Again, the dynamic conformational ensemble affects catalysis, a result that can aid the design of more efficient and processive Pols for amplifying damaged DNA (e.g., to convert a rare chemical damage event into a detectable genetic signal).^83^

While all the above mechanistic investigations mostly refer to Pol fidelity, we have begun computationally examining Pol processivity by considering the physical and dynamic steps of DNA translocation that occur at every Pol catalytic cycle following nucleotide addition (Figure 1A). The newly elongated double-stranded DNA has to shift along the Pol enzyme to recreate the initial configuration at the metal-aided reactive center.^85^ Here, based on structural data on Pol η, we could build multiple models that were simulated in the pre- and post-translocation states.^2^ In this way, we could show that the translocation mechanism (pre- to post-translocation) proceeds through enzyme motions in which Pol η advances asynchronously along the two DNA strands. Equilibrium molecular dynamics and deep-learning-guided enhanced-sampling simulations revealed that this mechanism relies on a set of positively charged residues that act in a coordinated manner at the Pol η–DNA interface. Through a motion reminiscent of windshield wipers, the dynamics of this set of selected, strategically located positively charged residues facilitate DNA translocation (Figure 1B and 1C). Such a coordinated dynamics allows these residues to grasp and slide the DNA substrate inside the Pol binding site.^2^ In this case, however, it was essential to capture these coordinated motions by leveraging machine-learning-based CVs,^86^ which enabled the collection of multiple transitions connecting pre- and post-translocation states (Figure 2A). This was a good example of where the timely advent of machine-learning CVs was truly instrumental, as such complex dynamics were difficult (and sometimes impossible) to investigate with a few standard and conventional RCs.^49,50^

**Figure 1:**
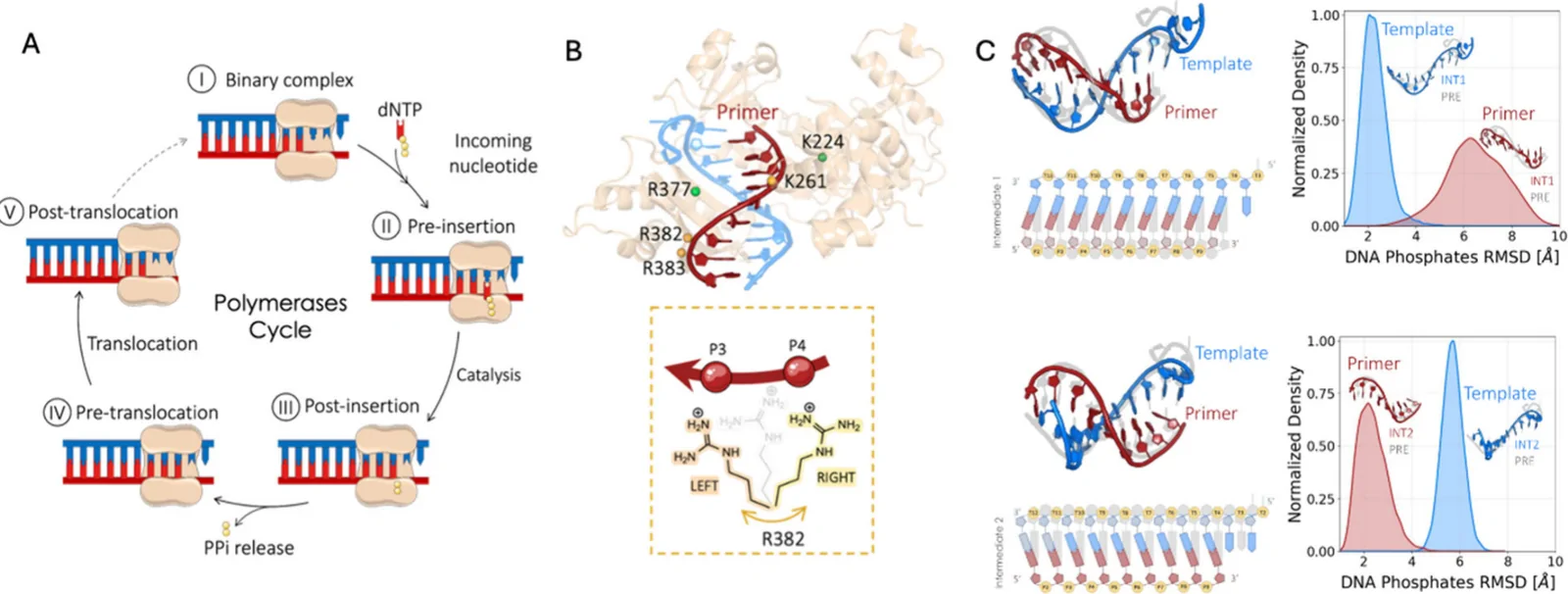
Coordinated residue motions to promote DNA translocation in polymerases. (A) Pol catalytic cycle. (B) Top:Positive residues at the Pol η·DNA interface, interacting with the primer strand (red); Bottom: motion of the R382 side chain, which transiently interacts with the primer strand. (C) Translocation in Pol η, where the primer strand is alternatively shifted by 1 base pair relative to the pre-translocation state, or vice versa. On the right, the probability distributions of RMSD values for DNA phosphates in the primer (red) and template (blue) strands, for the two possible translocation pathways.^2^ Adapted with permission from ref 2. Copyright 2025 American Chemical Society.

**Figure 2:**
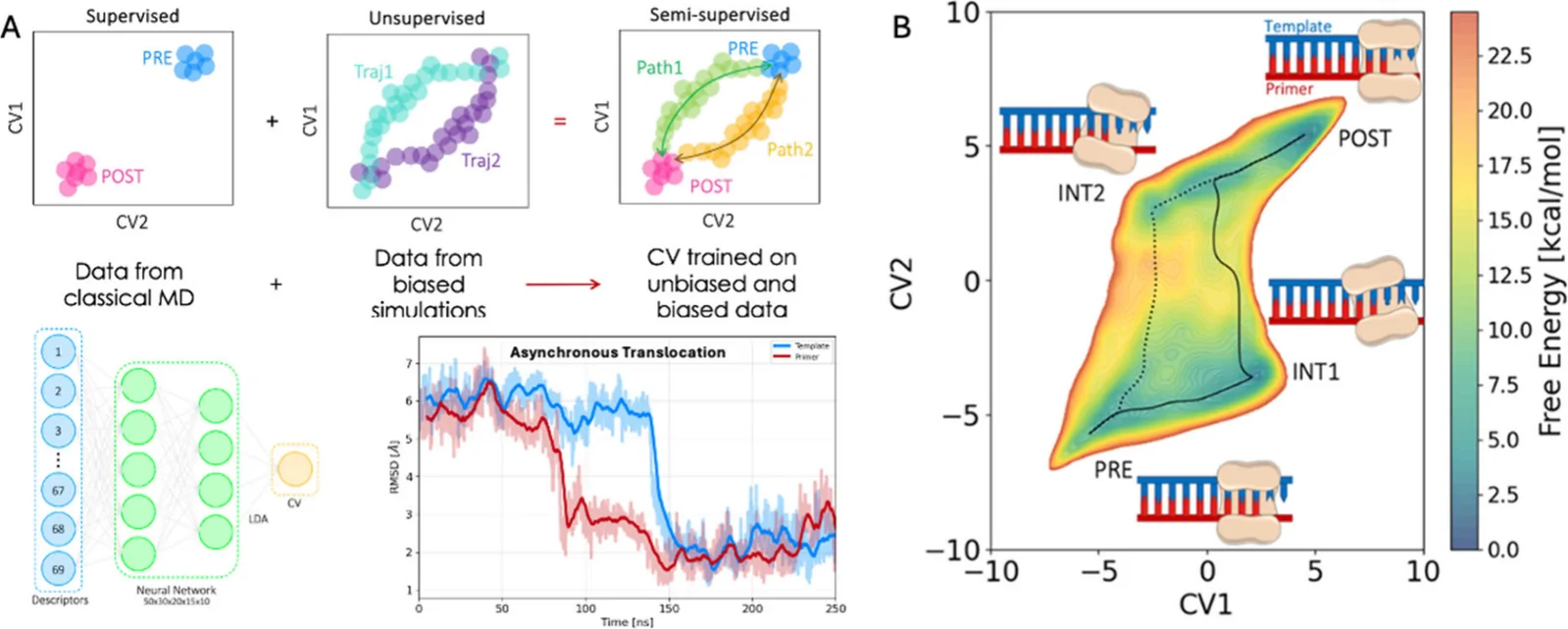
Machine-learning-based collective variables (CVs) for complex chemical processes. (A) Deep-LDA framework, where the CV is expressed using a neural network that takes as inputs a set of physical descriptors (features) and is optimized to maximally separate the metastable states. The training requires a data set of configurations from metastable states, along with their corresponding labels, which can be obtained by unbiased simulations from each state. The choice of descriptors is critical (*read paragraph in the main text*). To better describe two alternative reactive pathways, we designed a two-dimensional machine-learning CV, optimized in a semi-supervised multitask framework (Multi-Task CV). At the center, the RMSD of the template strand (blue) and the primer strand (red) over time during one translocation event, indicating an asynchronous mechanism. (B) Free energy surface (FES) describing the two translocation pathways.^2^ Adapted with permission from ref 2. Copyright 2025 American Chemical Society.

In more detail, we could train a classifier-based CV (Deep-LDA CV)^50^ using information limited to the metastable initial basins and drive initial exploratory transitions. Then, we exploited the newly generated simulation data, which suggested two alternative reaction pathways, to train a more informative two-dimensional CV using a multitask approach,^87^ thereby enabling the final production runs (Figure 2B). A cautionary note, however, is needed: While these sophisticated CVs are extremely helpful for elucidating complex multistep mechanisms, as explained in the initial methodological paragraph, much depends on the features used to generate them. Identifying the key features to generate optimal CVs requires bespoke analytical work on equilibrium MD trajectories. This key methodological aspect remains somewhat user-dependent and can be labor-intensive (in our experience), leaving ample room to make this human-in-the-loop machine-learning CV identification more effective, which partially explains the intense and incessant activity in this important field for CV-guided enhanced sampling simulations.

## Controlled Dynamics at the Catalytic Core of RNA Enzymes

Over the past decade, extensive atomistic multiscale molecular simulations have also been quite instrumental in investigating the catalytic core of RNA enzymes,^17,88,89^ such as the fundamental splicing reaction catalyzed by self-splicing group II introns,^3,90–92^ which are ribozymes evolutionarily and chemically related to the eukaryotic spliceosome. Based on pioneering structural data on these RNA enzymes,^93^ classical and quantum-based MD simulations could elucidate several key mechanistic aspects of RNA catalysis. Magistrato et al. elucidated, through QM­(Car–Parrinello)/MM simulations combined with thermodynamic integration, the molecular mechanism of cleavage of the 5′-exon, the first and rate-determining step of this splicing process.^90^ Results proposed a mechanism in which the nucleophilic water is activated before its attack on the RNA scissile phosphate.^90^ Using classical MD and QM/MM simulations, we later further clarified nucleophile activation, proposing that it occurs via the N3 atom of the conserved cytosine 358, which can act as a general base and accept the shuttled proton from the attacking water.^3^ This mechanistic step was then coupled with the clarification of an experimentally observed reversible structural rearrangement of the active site (i.e., the toggling state), which could be captured and explained through classical and enhanced-sampling MD simulations (Figure 3A–G).^90^ Also here, it was remarkable to observe the coordinated motions of some catalytic elements, including a transient potassium ion (K1), which was critical for regulating the active-site dynamics induced by the first splicing step, facilitating progression to the second step (Figure 3H,I).^3,78,94^ Notably, we have also demonstrated that small molecules can target this catalytic core to block splicing, opening a new way to modulate this fundamental reaction.^95^

**Figure 3:**
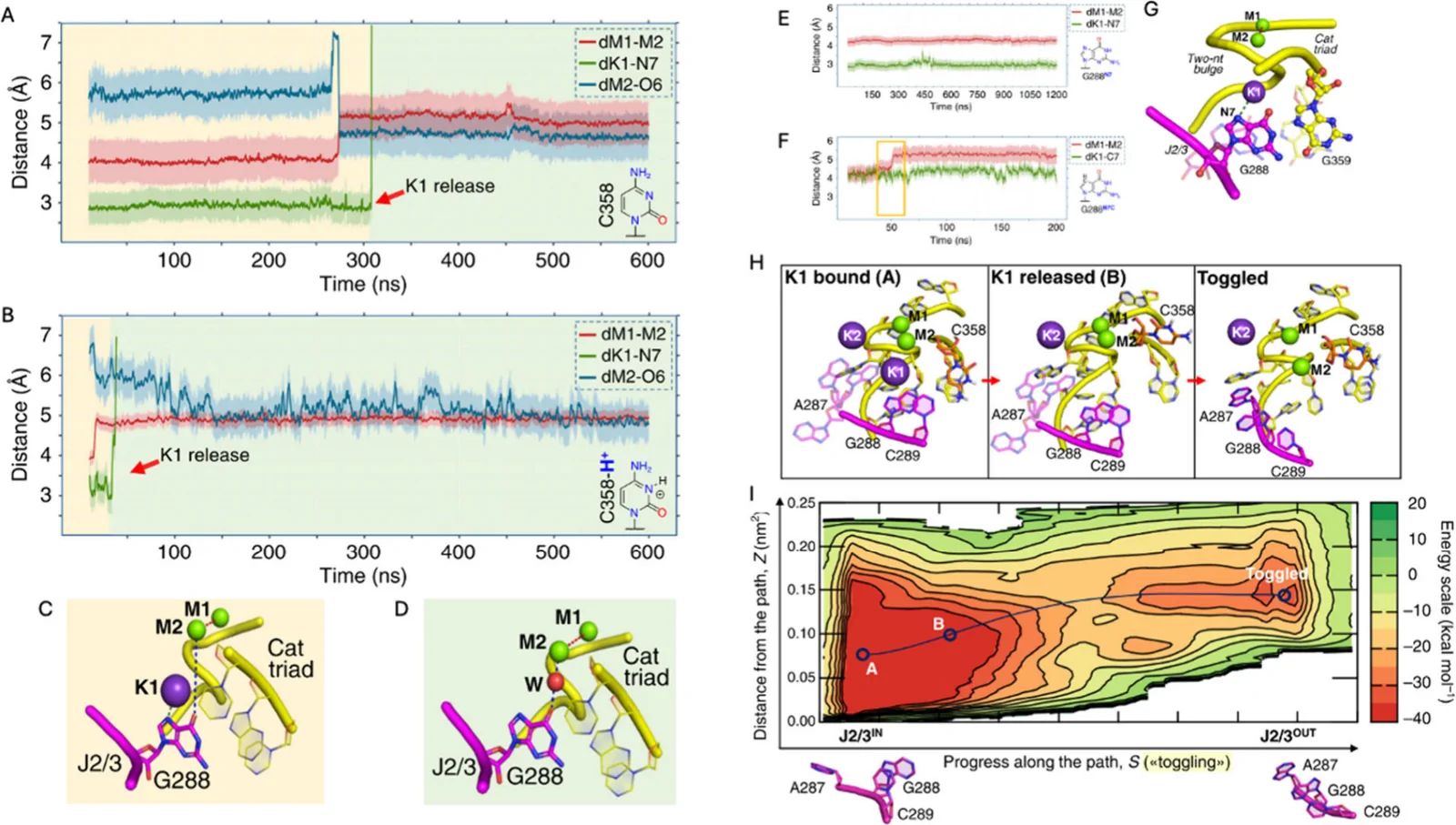
Visualizing group II intron dynamics between the first and second steps of splicing. (A-B) K1 release during MD simulations. (C–D) Structure of the active site at the beginning and at the end of the simulations, after K1 release. (E-F) Changes during MD simulations of the prehydrolytic state for key structural descriptors dK1-N7^G288^ (green trace) and dM1-M2 (red trace). (G) Representation of the K1–N7 interaction modeled from the simulations of the prehydrolytic state. (H) Active site for the intermediate A, B, and toggled states from metadynamics. (I) Free-energy landscape for toggling formation. The conformations of the J2/3 junction in state A and in the toggled state are represented.^3^ Adapted with permission from ref 3. Copyright 2020 Springer Nature.

These results and mechanistic insights into group II intron splicing parallel functional data on the spliceosome, reinforcing the notion that these evolutionarily related molecular machines share a common enzymatic strategy.^17^ In this regard, we have recently leveraged Cryo-EM data to clarify major conformational rearrangements of the megadalton spliceosome structure during splicing.^96–98^ We could simulate multiple splicing intermediates (>2 M atoms) to identify a minimal set of positively charged residues—evolutionarily conserved across species from human to yeast and associated with diseases if mutated—whose controlled dynamics coordinate with the transient, temporally ordered binding of spliceosomal proteins (Prp11, Prp8, and Yju2) at the spliceosome catalytic core (Figure 4A–D).^1^ This molecular mechanism ensures precise and timely spliceosome activation, confirming that the transient dynamic K1 and the timely positioning of the central catalytic metals and positively charged residues at the catalytic core are essential elements of the catalytic process (Figure 5A–C).^1^

**Figure 4:**
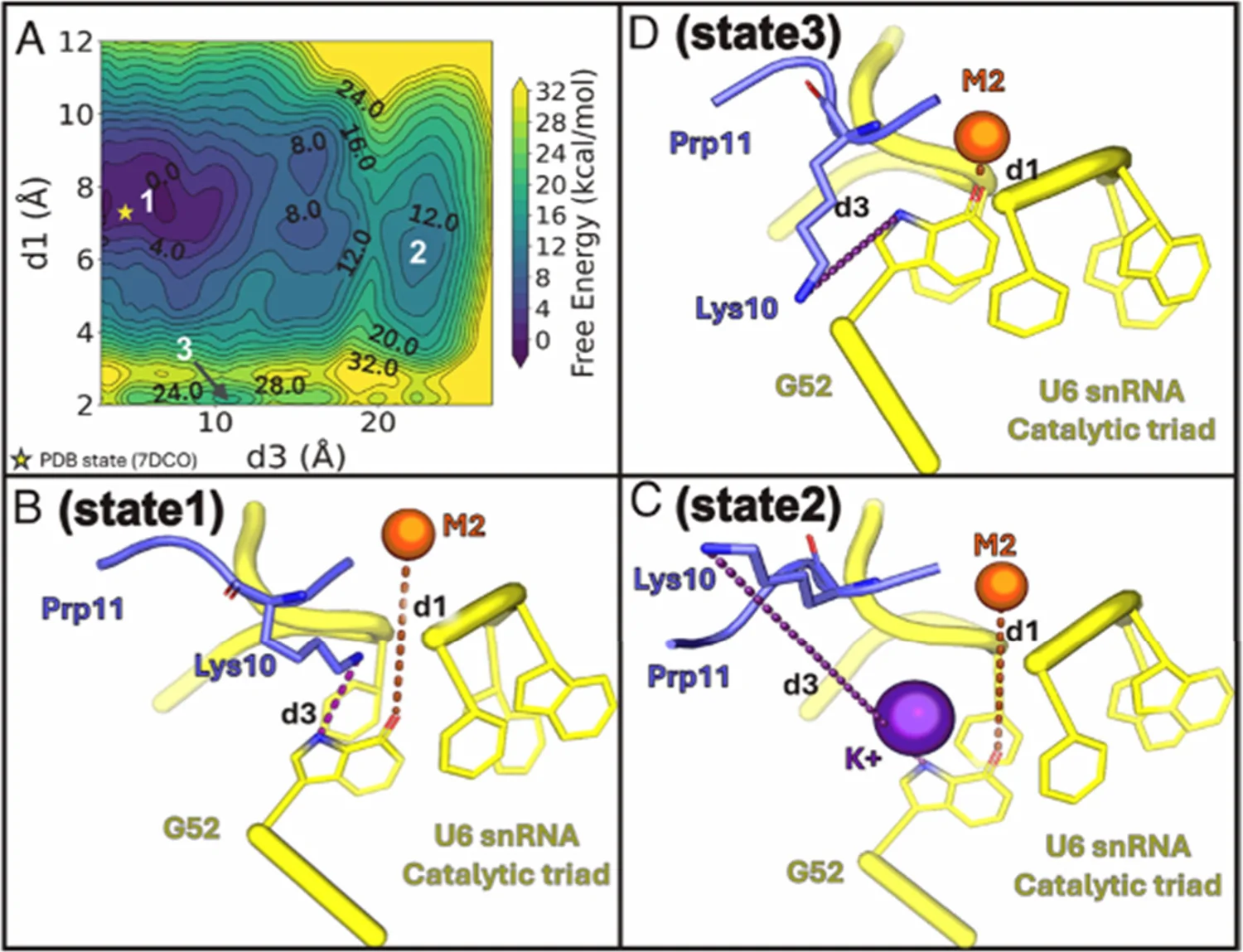
Controlled dynamics at the spliceosome catalytic core. (A) Energetics for the occupancy of the K1 site and the position of the M2 ion in the spliceosome.^1^ (B–D) Structures of three metastable states from metadynamics simulations. State 1 (B): a PDB-like arrangement where Lys10 remains at the K1 site. State 2 (C): Lys10 leaves the K1 site, allowing a K+ ion to occupy it and stabilize M2 in place. State 3 (D): M2 directly coordinates G52, partially occupying the K1 site. Adapted with permission from ref 1. Copyright 2026 National Academy of Sciences″.

**Figure 5:**
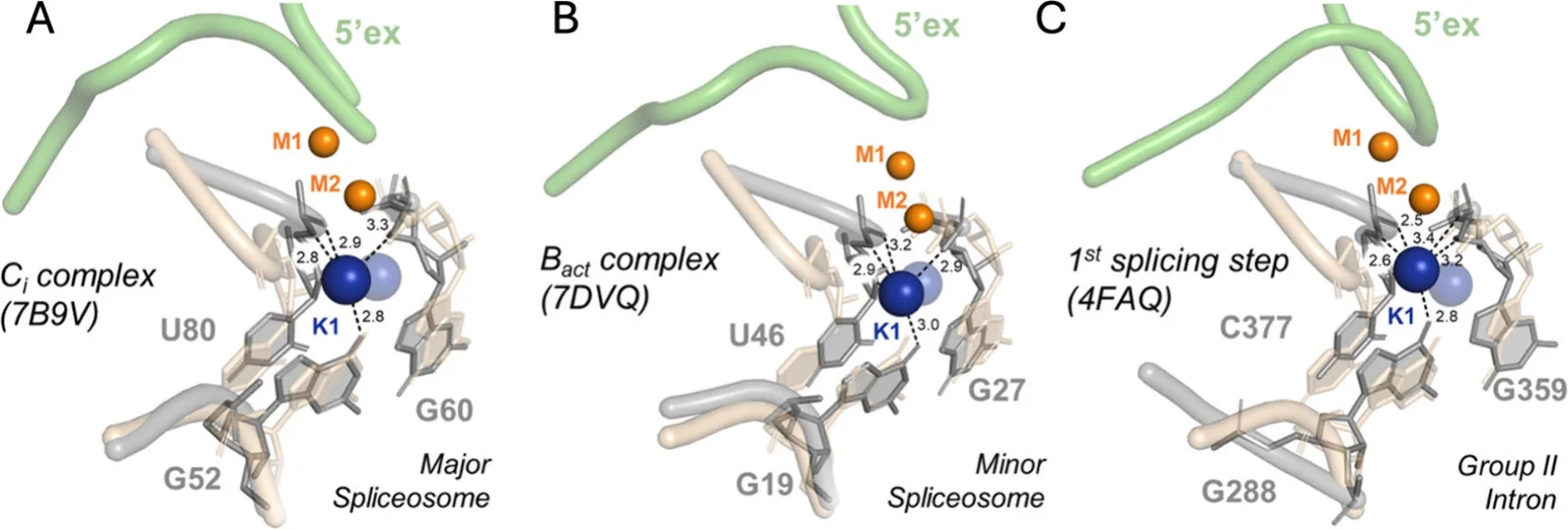
Prospective exploration of the spliceosome structure. Structural superposition of the K1 binding model over the spliceosome (panel A and B), and the group II intron (panel C). The predicted K1 ion (blue) is depicted as a semitransparent blue sphere.^17^ Adapted with permission from ref 17. Copyright 2018 Elsevier.

## Multiple Ions and Second-Shell Residues on the Move for Catalysis

Most nucleic acid-processing enzymes operate via the two-metal-ion mechanism,^99^ in which two cations, typically 2 Mg ions, are located at the catalytic center and are well coordinated in a shell composed of carboxylate groups (from aspartate or glutamate residues) and water molecules. In addition, the phosphodiester bridge of the nucleic acid is part of this first coordination shell, properly positioned to undergo catalysis.^100–104^ This recognized structural motif is recurrently found in most polymerases, nucleases, ribozymes, and topoisomerases.^52^ Yet, over the past few years, as anticipated by a few surprising structural findings and far-reaching computational hypotheses, additional metal ions have been observed to contribute to the overall catalytic process.^65,105^ These additional transient ions have been investigated through simulations in several specific cases by various research groups, consistently highlighting their catalytic role, which complements the core functional role of the two-magnesium-ion reactive center.^64,66,106,107^ One example is the clarification of the functional implications of the dynamic exchange of multiple K+ and Mg2+ ions at the RNase H1 reaction center,^94^ which are timely positioned at nonoverlapping locations near the active site at different stages of catalysis, and are crucial for both reactants’ alignment and leaving-group departure. Another notable example is the third catalytic ion, dynamically recruited from the bulk and promptly positioned near the catalytic center, as observed in the human exonuclease 1 (hExo1) metalloenzyme (Figure 6A-C)^108^ as reported by Magistrato et al. and by us in RNA enzymes.^3,107^ Recently, Palermo et al. investigated the similar trafficking and catalytic roles of metals in the context of CRISPR genome editing.^109^

**Figure 6:**
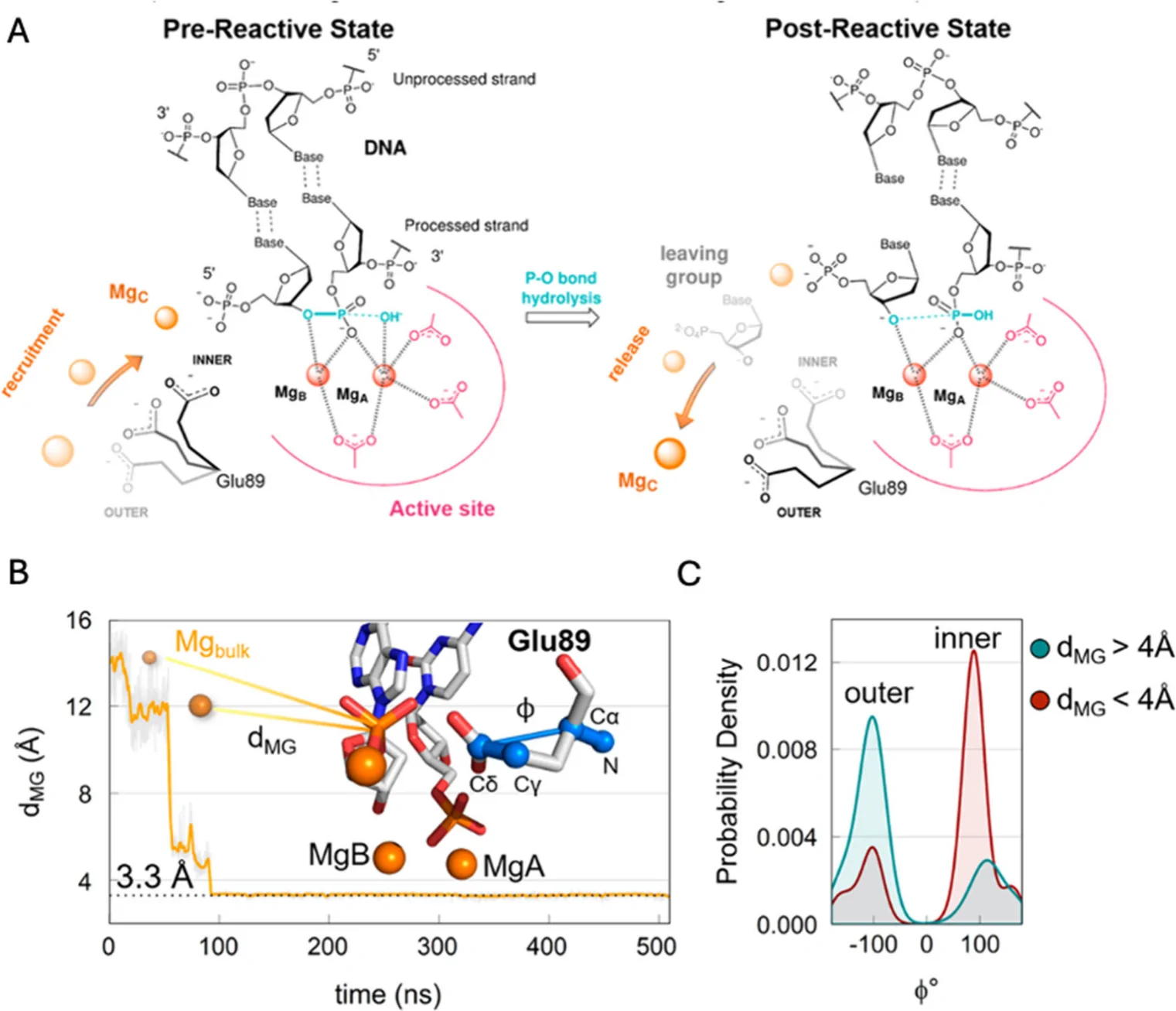
Dynamic recruitment of a transitory ion at the catalytic site in Exo1. (A) Motion of the transient third metal ion in Exo1, intermittently recruited/released by Glu89 (switching its inner/outer conformations) during catalysis.^108^ (B) Mg_bulk_ approaching the terminal 5′ phosphate. (B) Probability density of the location of Glu89 during the simulation: (blue) probability density for d_MG_ values >4 Å shows the outer conformation as the most populated; (red) probability density for d_MG_ values <4 Å shows the inner conformation is the most populated. Adapted with permission from ref 108. Copyright 2020 American Chemical Society.

Taken together, experimental and computational findings have therefore confirmed that, often, additional catalytic elements contribute to an expanded two-metal-ion architecture, as we first hypothesized based on structural observations and atomistic multiscale molecular simulations.^78^ Multiple basic amino acids and transient cations are functional parts of these outstanding enzymatic machines, occupying structurally conserved positions near the active site, as seen in several two-metal-ion enzymes, including polymerases, nucleases such as Cas9, and splicing ribozymes.^12,14,91,110^ Integrating structural analyses and atomistic multiscale molecular simulations is therefore key to a broader and nowadays prospective comprehension of these complex enzymatic machines in which function occurs through controlled dynamics.^17^

## Conclusions and Outlook

The critical examination of the body of work contained in this Account shows that atomistic multiscale molecular simulations nowadays offer prospective, experimentally supported mechanistic insights that will undoubtedly play an increasingly prominent role in the ongoing exploration of many other large macromolecular machineries essential to life and critically involved in disease, but difficult to experimentally characterize at high resolution. Such simulations can address the challenges posed by the model dimensions and functional complexity of ultralarge complex systems, which are often composed of millions of atoms explored via multimicrosecond dynamics that were previously prohibitive. These simulations help decode the coupling between conformational dynamics and chemical reactivity, explicitly linking the collective motions of specific and often conserved structural elements to catalytic steps in nucleic acid-processing enzymes. The mechanistic view that function is, essentially, dynamics—through the coordinated atomic motions that define function in these complex enzymes—adds a conceptual layer to the comprehension of these enzymatic processes. We have seen several cases where a specific, coordinated dynamics is, in fact, executed by complex enzymatic architectures that have evolved to actuate precise mechanisms as intricate and exacting as those found in high-end watchmaking. Continued learning about how these machines move-to-function at the atomistic scale will therefore certainly aid in the rational design and modulation of their action on RNA and DNA, paving the way for future breakthroughs in science and medicine.
